# Test - retest reliability of two instruments for measuring public attitudes towards persons with mental illness

**DOI:** 10.1186/1471-244X-11-11

**Published:** 2011-01-14

**Authors:** Bengt Svensson, Urban Markström, Ulrika Bejerholm, Tommy Björkman, David Brunt, Mona Eklund, Lars Hansson, Christel Leufstadius, Amanda Lundvik Gyllensten, Mikael Sandlund, Margareta Östman

**Affiliations:** 1 Department of Health Sciences, Lund University, PO Box 157 221 00 Lund, Sweden; 2 Department of social work, Umeå University 901 87 Umeå, Sweden; 3 Department of clinical sciences/Psychiatry, Umeå University 901 85 Umeå, Sweden; 4 Faculty of Health and Society Malmö University 205 06 Malmö, Sweden; 5 School of Health Sciences and Social Work Linaeus University Georg Lückligs väg 8, 351 95 Växjö, Sweden

## Abstract

**Background:**

Research has identified stigmatization as a major threat to successful treatment of individuals with mental illness. As a consequence several anti-stigma campaigns have been carried out. The results have been discouraging and the field suffers from lack of evidence about interventions that work. There are few reports on psychometric data for instruments used to assess stigma, which thus complicates research efforts. The aim of the present study was to investigate test-retest reliability of the Swedish versions of the questionnaires: FABI and "Changing Minds" and to examine the internal consistency of the two instruments.

**Method:**

Two instruments, fear and behavioural intentions (FABI) and "Changing Minds", used in earlier studies on public attitudes towards persons with mental illness were translated into Swedish and completed by 51 nursing students on two occasions, with an interval of three weeks. Test-retest reliability was calculated by using weighted kappa coefficient and internal consistency using the Cronbach's alpha coefficient.

**Results:**

Both instruments attain at best moderate test-retest reliability. For the Changing Minds questionnaire almost one fifth (17.9%) of the items present poor test-retest reliability and the alpha coefficient for the subscales ranges between 0.19 - 0.46. All of the items in the FABI reach a fair or a moderate agreement between the test and retest, and the questionnaire displays a high internal consistency, alpha 0.80.

**Conclusions:**

There is a need for development of psychometrically tested instruments within this field of research.

## Background

Negative public attitudes towards persons suffering from mental illness have been identified as an obstacle for recovery from illness and becoming full participants in society [1]. The concept of stigmatization has been described and operational definitions have been made by various researchers [2]. Although there is no clear consensus, stigmatization of people with mental illness is usually described as a complex of problems emanating from a lack of knowledge about mental health problems, negative attitudes and excluding or avoiding behaviours towards individuals suffering from mental health problems. For those who are subjected to stigma and their relatives this leads to a perception of the public as ignorant, prejudiced and discriminating in their view of mental illness [1].

It has been shown that stigmatization reduces life chances for persons suffering from mental illness. The negative effects include less access to mental health services and to advances in psychiatric treatment [3,4], to psychosocial stress, to low socio-economic status and to delay in appropriate help seeking [5]. Relatives of mentally ill persons identify stigma as a key problem that causes their ill relatives a lowered self-esteem, difficulties in making and keeping friends, and a tendency to deny that they have a mental illness [6]. The relatives also experience burden due to the stigma associated with mental illness. In a study investigating family stigma one group of relatives reported thoughts of life and death in terms of their relatives and themselves.. Eighteen per cent of the relatives had sometimes felt that the patient would have been better off dead. Ten per cent of the family members in the same study reported that they now and then had experienced suicidal thoughts [7].

Persons with a diagnosis of mental disorder also present difficulties in seeking and keeping work. In a study of people suffering from schizophrenia in five European countries the employment rate ranged from 5% (England) to a maximum of 23% (Italy) [8]. One explanation for the low employment rate is that employers discriminate against persons who have a history of psychiatric treatment. In a study of 200 Human Resource Officers in UK companies it was shown that a history of mental illness significantly reduced the chances of employment in comparison with a history of diabetes [9]. Two studies in California found that employers perceived people with mental illness as being as undesirable as ex-convicts and that the only group they were more reluctant to hire were people with active tuberculosis [10].

To suffer from mental health problems also leads in many cases to a process of identity transformation where persons lose their previously held or desired identities and adopt a stigmatised view of themselves. This process is referred to as self-stigma [11], internalised stigma [12] or anticipated stigma [13]. Self-stigma has been associated with reductions in protective psychological variables such as hope [14], self-esteem [15,16], self-efficacy [17], empowerment [17,18] and recovery beliefs [18]. It is also associated with lower quality of life [15,17] and an increase in avoidant coping strategies such as withdrawal and secrecy.

Several programs to fight stigma have been initiated as a consequence of the research findings that have indicated stigmatization as a threat to successful treatment and rehabilitation of individuals with mental illness. Some of these programs have been international, such as the World Psychiatric Association's Global Programme against Stigma and Discrimination because of Schizophrenia [19], others have been national such as the Changing Minds Campaign by the Royal College of Psychiatrists in the United Kingdom [20,21], the PSYKE-Campaign in Sweden [22] and the National Mental Health charity SANE Australia [23]. The overall ambitions have been to increase public knowledge about mental illness and its treatment possibilities, to create more positive attitudes towards people with mental illness and to reduce prejudice and discrimination. To some extent the aims of these campaigns have been achieved [19,24]. However, despite all efforts to reduce stigma through public campaigns the results are not convincing and the field suffers from a lack of evidence about what works [1]. The process appears to be a slow one. In a recent study 73% of a cohort of persons suffering from mental illness believed that most employers would pass over an application from a former psychiatric patient in favour of another applicant and that they are not considered as trustworthy as the average citizen (67%) or as intelligent as other people (56%) [25]. In another recent study a list of 250 derogatory labels used among fourteen year old schoolchildren to stigmatize people with mental illness were described. The authors suggest that this wide-ranging and negative number of terms about mental illness appears to emanate from the media and from family and peers [26]. All these findings indicate a quite persistent negative attitude in the general population.

There is a need for further work in the struggle against stigma and valid and reliable instruments need to be tested in order to measure effects of interventions. Link et al [27] have reviewed different methods for measuring mental health stigma but there is little information about psychometric properties of the instruments used in the various studies. There is often information about the internal consistency of the different scales but nothing about their test-retest reliability. The reliability in this respect is crucial when using the scales to evaluate the rate of success associated with different interventions. The present study was carried out to determine psychometric properties of two instruments used in earlier studies. The instruments chosen were: "The self-report inventory of fear of and behavioural intentions toward the mentally ill (FABI) [28,29] and the set of questions used in the campaign "Changing Minds: Every Family in the Land" initiated by the Royal College of Psychiatrists [20]. The purpose was to create Swedish versions of instruments that could be used in research on attitudes towards persons with mental illness. Being as no data concerning psychometric properties concerning these scales have been presented it is important to conduct a trial to establish this information.

## Aims

The aims of the study were:

- to investigate test-retest reliability of the Swedish versions of the self-report inventory: FABI and of the "Changing Minds"- questionnaire

- to examine the internal consistency of the two instruments

## Methods

Permission to translate the instruments was obtained from the original authors. The instruments were first translated into Swedish and then retranslated into English. The language in the different versions were compared and adjusted to the final Swedish versions.

### The questionnaires

The self-report inventory of fear of and behavioural intentions toward the mentally ill (FABI) is a ten item questionnaire with a five point response scale for each question. Responses are coded from 1 to 5 and the items include:

Fear: (response alternatives: strongly agree, agree, neutral, disagree and strongly disagree)

"I am afraid of people with mental illness"

Behavioural intentions: (response alternatives: very likely, likely, uncertain, unlikely, very unlikely)

"Would you object to having mentally ill people living in your neighbourhood?"

"Would you avoid conversations with neighbours who had suffered from mental illness?"

"Would you be willing to work with somebody with a mental illness?"

"Would you invite somebody into your home if you knew they suffered from mental illness?"

"Would you be worried about visiting somebody with a mental illness?"

"If somebody had been a former psychiatric patient, would you have them as a friend?"

"If somebody who had been a former psychiatric patient came to live next door to you, would you greet them occasionally?"

"Would you have casual conversations with neighbours who had suffered from mental illness?"

"If somebody who had been a former psychiatric patient came to live next door to you, would you visit them?" [28,29]

### Changing Minds: Every family in the Land

The instrument was, in the original study, administered as an interview and two sets of data were obtained [20]. The first set collected data about household composition and individual demographic and employment-related circumstances. This set was not used in the present study. The second set was designed to obtain perceptions about seven mental disorders, identified in pre-interview focus groups and then targeted by the Royal College of Psychiatrists campaign. The focus groups had indicated that these disorders were familiar to the general public. The disorders were severe depression, panic attacks, schizophrenia, dementia, eating disorders, alcoholism and drug addiction. The questions about the disorders were based on findings presented in a literature review on stigmatization of people with mental disorders by Hayward and Bright [30]. The key perceptions of persons with mental illness are that they are dangerous, unpredictable, are difficult to talk with, have only themselves to blame, are able to pull themselves together, have a poor outcome and don't respond very much to treatment according to this review. For each disorder the respondents could rank their perception on a five point scale from one extreme e.g. "dangerous to others", to the opposite, "not dangerous to others", "unpredictable" to "not unpredictable" and so forth. In the present study the questions were administered as a self-rating questionnaire. Information about the activities and the evaluation of the "Changing minds" campaign can be found on the website: http://www.rcpsych.ac.uk/campaigns/previouscampaigns/changingminds.aspx

### Sample and Data collection

The sample consisted of 51 students in a three year educational programme in nursing. They were in their second semester and during the data collection period they were studying physiology and anatomy. Nothing in their present education had, to our knowledge, a content that could affect their view of mental disorders. The first data collection was made in September, 2007 and the retest data collection was made three weeks later. No major media discussions concerning mental illness occurred during the time period between the test and retest data collection. Being as this investigation is a part of a larger study concerning the impact of formal education in psychiatry on attitudes towards mental illness baseline data from a larger database was used to examine internal consistency. This larger database consisted of 1001 students from educational programmes including psychiatry in their curriculum. The following student categories were represented: nursing, medicine, social work, physiotherapy, occupational therapy, psychology, public health work, and police trainee.

### Statistics

Weighted kappa coefficients were calculated for each item in the two questionnaires in order to investigate the test - retest reliability for the different instruments. The reference values for the strength of agreement are from Altman [31] who considers < 0.20 as poor agreement, 0.21-0.40 as fair, 0.41-0.60 as moderate, 0.61-0.80 as good and 0.81-1.00 as a very good agreement. Weighted kappa is used because it takes into account the magnitude of the discrepancy in ordinal data. A weakness of ordinary kappa statistics is that it treats all disagreements equally whether they are very large or very small [31]. Internal consistency was examined by calculating Cronbach's alpha coefficient for the FABI. In the questionnaire from the Changing Minds campaign the Cronbach's alpha coefficient was calculated for the responses concerning each disorder. The statistical software used was SPSS 15.0 and Vasserstats [32].

### Ethical considerations

In accordance with Swedish legislation research that not includes interventions does not require formal approval from a research ethic committee. The methods of evaluation conformed to the Helsinki Declaration.

## Results

The results from the "Changing minds" questionnaire display at best a moderate agreement between the test and retest. The items concerning unpredictability and whether it is difficult or not to talk to a person with a certain disorder appear in particular to have low test-retest reliability. The calculation of Cronbach's alpha shows that none of the batteries of questions about the different disorders has a sufficient level of internal consistency in order to be considered as scales (table 1).

**Table 1:** Weighted kappa and Cronbach alfa for the "Changing minds" questionnaire (95%Cls), (n = 51)

Opinion			Type of mental illness				
	**Severe depression**	**Panic attacks**	**Schizophrenia**	**Dementia**	**Eating disorder**	**Alcohol addiction**	**Drug addiction**

Danger to others	0.18 (0.01-0.36)	0.32 (0.14-0.51)	0.41 (0.21-0.61)	0.43 (0.27-0.60	0.32 (0.00-0.66)	0.17 (0.01-0.33)	0.25 (0.07-0.43)
Unpredictable	0.05 (0-0.22)	0.34 (0.14-0.53)	0.33 (0.08-0.58)	0.24 (0.05-0.42)	0.20 (0.00-0.41)	0.12 (0.00-0.30)	0.37 (0.18-0.57)
Hard to talk to	0.13 (0.00-0.35)	0.20 (0.02-0.39)	0.18 (0.00-0.38	0.42 (0.22-0.62)	0.27 (0.09-0.46)	0.27 (0.07-0.46)	0.31 (0.13-0.49)
Selves to blame	0.43 (0.25-0.62)	0.36 (0.17-0.55)	0.35 (0.14-0.55)	0.58 (0.29-0.86)	0.60 (0.46-0.74)	0.52 (0.34-0.70)	0.48 (0.31-0.66)
Not improved if treated	0.28 (0.04-0.52)	0.19 (0.00-0.45	0.48 (0.33-0.64)	0.38 (0.20-0.55)	0.23 (0.03-0.42)	0.12 (0.00-0.32)	0.44 (0.24-0.63)
Feel different	0.35 (0.15-0.55)	0.42 (0.23-0.62)	0.52 (0.27-0.77)	0.42 (0.18-0.65)	0.45 (0.24-0.65)	0.40 (0.22-0.58)	0.49 (0.29-0.68)
Pull self together	0.51 (0.32-0.69)	0.43 0.26-0.61)	0.38 (0.18-0.57)	0.32 (0.07-0.57)	0.42 (0.25-0.60)	0.45 (0.28-0.61)	0.31 (0.13-0.50)
Never recover	0.26 (0.08-0.45)	0.47 (0.23-0.71)	0.31 (0.12-0.49)	0.22 (0.02-0.42)	0.43 (0.18-0.69)	0.32 (0.12-0.52)	0.28 (0.18-0.56)

Cronbach alfa	0.26	0.40	0.27	0.21	0.19	0.41	0.46

Weighted kappa, < 0.20 poor agreement, 0.21-0.40 fair, 0.41-0.60 moderate, 0.61-0.80 good, 0.81-1.00 very good (Altman, 1991)

The complete "Changing minds" questionnaire consists of 56 items and 10 of these (17.9%) present poor test-retest reliability, 26 items (46.4%) fair and finally 20 items (35.7%) moderate test-retest reliability.

The weighted kappa values for the self-report inventory of fear and behavioural intentions towards the mentally ill (FABI) also attain at best moderate test-retest reliability. However, none of the items fall below a fair agreement level between the test and retest. The questionnaire shows high internal consistency (table 2).

**Table 2:** Weighted kappa and Cronbach alfa for the self-report inventory of fear of and behavioural intentions toward the mentally ill (95%Cls), (n = 51)

Item	Weighted kappa (95% Cls)
I am afraid of people with mental illness	0.37 (0.18-0.56)
Would you object to having mentally ill people living in your neighbourhood?	0.29 (0.13-0.46)
Would you avoid conversations with neighbours who had suffered from mental illness?	0.41 (0.21-0.60)
Would you be willing to work with somebody with a mental illness?	0.31 (0.12-0.49)
Would you invite somebody into your home if you knew they suffered from mental illness?	0.54 (0.36-0.72)
Would you be worried about visiting somebody with a mental illness?	0.28 (0.06-0.50)
If somebody had been a former psychiatric patient, would you have them as a friend?	0.36 (0.09-0.63)
If somebody who had been a former psychiatric patient came to live next door to you, would you greet them occasionally?	0.31 (0.06-0.57)
Would you have casual conversations with neighbours who had suffered from mental illness?	0.41 (0.18-0.64)
If somebody who had been a former psychiatric patient came to live next door to you, would you visit them?	0.30 (0.11-0.49)
Cronbach alfa	0.80

Weighted kappa, < 0.20 poor agreement, 0.21-0.40 fair, 0.41-0.60 moderate, 0.61-0.80 good, 0.81-1.00 very good (Altman, 1991)

## Discussion

The investigation of the test-retest reliability in attitudes towards the mentally ill during a three week period indicates a certain level of instability in attitudes as measured with the "Changing minds" questionnaire and FABI. The sample consisted of nursing students studying in their first year of the educational programme. No demographic data for the group investigated was collected but a fair estimation is that it consists of a majority of females with a stable middle class background and with a mean age between 20 and 25. They can thus not be considered to be representative for any group other than nursing students. There are, however, no plausible hypotheses that state that a group consisting of a majority of young women should be more inconsistent in their attitudes than any other group. The fact that the students were trainees in a helping profession might be of interest. If they, after the first completion of the questionnaire, became interested in the issues presented and found out more about these then this may have contributed to a change in their attitudes. Though, health care professionals have in several studies shown attitudes even less favourable than members of the general public [33-35].

Comparisons are difficult to make being as no previous psychometric data for the instruments have been published. The internal consistency reliability of scales measuring social distance tends to be good or excellent [27], which is also repeated for the self-report inventory of fear of and behavioural intentions towards the mentally ill (FABI) in the present study. The questions in FABI deal with intentions that to a high degree reflect social distance, so in that respect the results are quite consistent with earlier findings.

The "Changing minds" questionnaire displays poor internal consistency reliability and rather problematic overall test-retest reliability. This is quite unexpected; the scale has high face validity and is based on ambitious research [20]. A similar technique to construct questionnaires was used by Olmsted and Durham [36]. They studied attitudes among college students towards the mentally ill using a scale containing pairs of adjectives representing opposites, such as "valuable - worthless, clean - dirty, predictable - unpredictable, etcetera". The results from analyses of three different cohorts, sociology students from 1962, from 1971 and a stratified sample of "general public" show a very high correlation in attitudes between both groups and time points. This indicates a high stability in attitudes at a group level. Whether this also mirrors high test-retest reliability cannot be deduced due to the lack of data. The fact that the "Changing minds" questionnaire was used as a self-rating instrument and the information in the original studies was obtained through interviews might make a difference. It is, however, difficult to imagine the potential significance of this difference being as no comparable data exist. The research of stigma towards the mentally ill has increased and a number of different instruments are in use [27]. Further research is thus needed to create psychometrically sound instruments for securing valid and reliable results.

## Conclusion

Psychometrically tested instruments for measuring public attitudes towards persons with mental illness are generally lacking. The present study indicates low test-retest reliability for the two scales investigated. To ensure the usefulness of further research within this particular research field a development of reliable and valid measurements is needed.

## Competing interests

The authors declare that they have no competing interests.

## Authors' contributions

All authors were involved in the design of the study and in the data collection for the larger study used for establishing the internal consistency of the scales. BS and TB made the data collection for the test-retest investigation. BS analyzed the data and wrote the manuscript together with MÖ and UM. All authors read and approved the final manuscript.

## Pre-publication history

The pre-publication history for this paper can be accessed here:

http://www.biomedcentral.com/1471-244X/11/11/prepub
